# Activation of Aryl Hydrocarbon Receptor (AhR) Leads to Reciprocal Epigenetic Regulation of FoxP3 and IL-17 Expression and Amelioration of Experimental Colitis

**DOI:** 10.1371/journal.pone.0023522

**Published:** 2011-08-15

**Authors:** Narendra P. Singh, Udai P. Singh, Balwan Singh, Robert L. Price, Mitzi Nagarkatti, Prakash S. Nagarkatti

**Affiliations:** 1 Department of Pathology, Microbiology and Immunology, University of South Carolina School of Medicine, Columbia, South Carolina, United States of America; 2 Primate Research Center, Emory University, Atlanta, Georgia, United States of America; University of California San Francisco, United States of America

## Abstract

**Background:**

Aryl hydrocarbon receptor (AhR), a transcription factor of the bHLH/PAS family, is well characterized to regulate the biochemical and toxic effects of environmental chemicals. More recently, AhR activation has been shown to regulate the differentiation of Foxp3^+^ Tregs as well as Th17 cells. However, the precise mechanisms are unclear. In the current study, we investigated the effect of 2,3,7,8-tetrachlorodibenzo-p-dioxin (TCDD), a potent AhR ligand, on epigenetic regulation leading to altered Treg/Th17 differentiation, and consequent suppression of colitis.

**Methodology/Principal Findings:**

Dextran sodium sulphate (DSS) administration induced acute colitis in C57BL/6 mice, as shown by significant weight loss, shortening of colon, mucosal ulceration, and increased presence of CXCR3^+^ T cells as well as inflammatory cytokines. Interestingly, a single dose of TCDD (25 µg/kg body weight) was able to attenuate all of the clinical and inflammatory markers of colitis. Analysis of T cells in the lamina propria (LP) and mesenteric lymph nodes (MLN), during colitis, revealed decreased presence of Tregs and increased induction of Th17 cells, which was reversed following TCDD treatment. Activation of T cells from AhR^+/+^ but not AhR ^-/-^ mice, in the presence of TCDD, promoted increased differentiation of Tregs while inhibiting Th17 cells. Analysis of MLN or LP cells during colitis revealed increased methylation of CpG islands of Foxp3 and demethylation of IL-17 promoters, which was reversed following TCDD treatment.

**Conclusions/Significance:**

These studies demonstrate for the first time that AhR activation promotes epigenetic regulation thereby influencing reciprocal differentiation of Tregs and Th17 cells, and amelioration of inflammation.

## Introduction

Aryl hydrocarbon receptor (AhR) is a transcription factor that resides in the cytosol, and is a member of the bHLH-PAS protein family [1], [2]. Activation of AhR leads to conformational change and translocation to the nucleus where it binds to its dimerization partner, aryl hydrocarbon receptor nuclear translocator (ARNT). The AhR-ARNT complex initiates transcription of genes with promoters containing a dioxin-responsive element (DRE) consensus sequence. Wide range chemicals can activate AhR including environmental contaminants such as 2,3,7,8-Tetrachlorodibenzodioxin (TCDD), and other compounds such as tryptophan derivatives, flavonoids and biphenyls [3]. AhR was first discovered and well characterized as a transcription factor responsible for the activation of genes encoding a number of xenobiotic metabolizing enzymes, and mediate the toxicity induced by TCDD [4]. Interestingly, recent studies indicated that AhR activation plays diverse roles in cellular functions, including the regulation of the immune system [5], [6].

Recently, the role of AhR activation in the regulation of T cell differentiation has generated significant interest [7], [8]. AhR was shown to be express by both Th17 and Tregs, and furthermore, AhR activation promoted their differentiation [9], [10]. The development of Tregs and Th17 cells is reciprocally regulated. Thus, while transforming growth factor (TGF)-β1 induces the differentiation of Treg cells [11], the TGF-β1 along with combination of IL-6 or IL-21 results in the differentiation of Th17 [12], [13]. Thus, it is not clear how AhR activation leads to the induction of both Tregs and Th17 cells. One possibility is that the type of ligand and its affinity to AhR may determine whether Th17 or Tregs are induced [14]. Moreover, previous studies from our laboratory have shown that activation of AhR by TCDD induces up-regulation of Fas and Fas ligand, thereby promoting activation-induced cell death [15], [16], [17]. Thus, *in vivo* these events are also likely to skew the T cell differentiation. The impact of such factors on T cell differentiation, *in vivo* has not been taken into consideration.

The inflammatory bowel disease (IBD), Crohn's disease (CD) and ulcerative colitis (UC), are syndromes characterized by chronic inflammation in gastrointestinal tract. Currently it is believed that persistent intestinal inflammation may be the result of a dysregulated immune response to commensal enteric bacterial antigens [18], [19]. The chronic nature of inflammation appears to be a polarized dysregulated immune response due to imbalance of immune homeostasis involving Th1, Th17, and/or Th2 cells [20], [21], [22], [23]. In addition, specialized population of regulatory T cells (Tregs) that express forkhead transcription factor (Foxp3) play an important role in the control of intestinal inflammation [24]. The naturally arising CD4^+^ CD25^+^ Tregs have been shown to prevent or even cure colitis in the T cell transfer model [25], [26]. Also, mutations in Foxp3 results in fatal immune disorder characterized by an uncontrolled T cell proliferation, and drastically elevated production of Th1 and Th2 cytokines suggesting the critical role played by Tregs in immune system homeostasis [27].

Oral consumption of DSS triggers colitis in mice that is similar in the expression profiles of cytokines as well as histological changes as those observed in human IBD, particularly UC [28]. Recent studies indicated that during acute DSS-induced colitis, there was induction in Th1 and Th17 cytokine profiles and that this converted into a Th2-biased (IL-4 and IL-10) inflammatory profile during the chronic stages of colitis [28], [29]. Mice deficient in IL-17A gene, showed only faint manifestations of colitis thereby suggesting that IL-17A plays a pivotal role in the pathogenesis of DSS-induced colitis [30]. In the current study, we used TCDD to activate the AhR and determine the reciprocal regulation of Th17 and Treg differentiation during DSS-induced colitis. We noted that TCDD ameliorated colitis by up-regulating Tregs and down-regulating Th17 cells through epigenetic regulation.

## Results

### Effect of AhR activation on DSS-induced colitis

Acute colitis was induced by using dextran sulfate sodium (DSS) as described elsewhere [31]. In brief, 8-week-old C57BL/6 mice received either drinking water or water containing 3% DSS (MP Biomedicals, LLC, Aurora, OH) (ad libitum) for 7 days followed by water cycle alone for 7 days. The body weight of mice was monitored every day from day 0, at the start of TCDD treatment. Mice received a single dose of 25 µg/kg body weight of TCDD dissolved in corn oil or corn oil alone as a vehicle control. Throughout the study, we used 4 groups of mice: Vehicle alone, TCDD alone, DSS+vehicle and DSS+TCDD.

**Figure 1:**
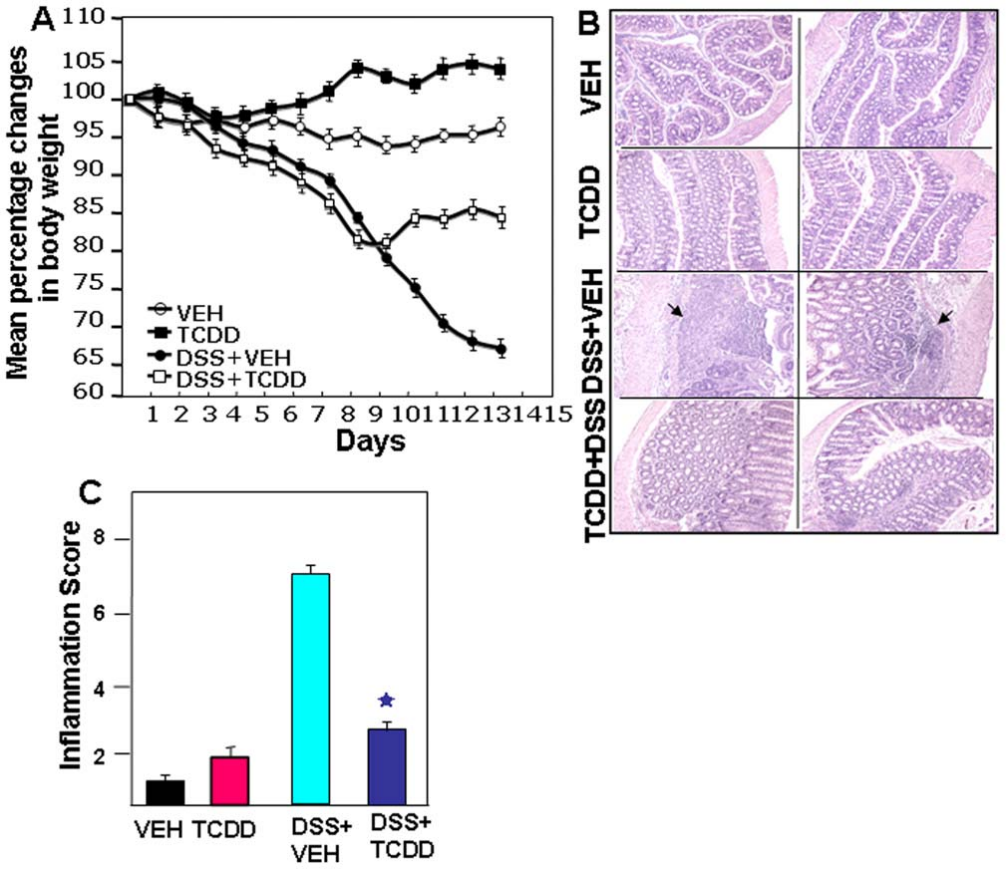
Change in body weight, histological characterization, and severity of colitis in mice after DSS induction and TCDD treatment. A. We used 4 groups of C57BL/6 mice: those that received VEH alone, TCDD (25 µg/kg body weight) alone, DSS+VEH, or a combination of DSS+TCDD. In DSS mice, TCDD was given as a single dose on the first day of DSS exposure. After seven days, DSS was replaced with a water cycle (*ad libitum*) for another seven days. The body weight of the mice was recorded daily. The statistical significance difference between each groups were assessed by using Mann-Whitney U Test. Data represents the mean of three experiment involving 6 mice per group. There was statistically significant difference between DSS+VEH vs DSS+TCDD groups (p<0.05). B. Histological sections of colons from the 4 groups of mice, DSS+VEH-treated mice showed significant lymphocyte infiltration and distortion of glands, while DSS+TCDD-treated mice showed colon lumen having markedly decreased lymphocyte infiltration. Other pathologic changes during DSS-induced colitis included diffuse leukocyte infiltrates, distorted crypts, and thickening of the lamina propria in the area of distorted crypts in the colon (B). These changes were significantly reversed in DSS+TCDD groups as seen in inflammation score (C). Asterisks indicate statistically significant differences; i.e., *p*<0.01 between DSS+VEH versus DSS+TCDD treated group.

DSS administration induced acute colitis in C57BL/6 mice, as shown by significant weight loss, shortening of colon, mucosal ulceration and increased inflammatory cytokine production. DSS-induced colitis caused significant reduction in the body weight, which continued to decline throughout the study (Fig 1A). Interestingly, a single dose of TCDD was able to reverse the course of the disease beginning day 9. Throughout the study, mice receiving TCDD alone showed no significant alterations in the various parameters such as inflammation score and inflammatory cytokines when compared to vehicle-treated mice. Thus, we have primarily focused our discussion below, on results comparing DSS+vehicle versus DSS+TCDD groups.

### AhR activation attenuates the severity of colitis

The colitis-induced mice that received TCDD had significant reductions in intestinal inflammation (Fig 1B). The mean histological scores in DSS+vehicle groups were significantly higher than the scores in mice treated with DSS+TCDD (Fig 1C). The pathologic changes associated with colitis included epithelial disruption, transmural necrosis, edema, and diffuse leukocyte infiltrates (polymorphonuclear leukocytes, lymphocytes and eosinophils) in the colon. The architecture of the crypts was distorted and the lamina propria was thickened in the area of distorted crypts. Most importantly, the number of inflammatory infiltrates was significantly reduced after TCDD treatment (Fig. 1B).

### AhR activation decreases the induction of serum inflammatory cytokine/chemokines associated with colitis

TNF-α, IFN-γ, MCP-1, IL-17, KC and Eotaxin-1 have been shown to be over-produced during inflammatory diseases, including IBD or in experimental model of colitis [32], [33], [34]. We therefore determined if TCDD treatment in DSS-induced colitis, would lead to decreased systemic levels of these cytokines and chemokines. DSS significantly increased these pro-inflammatory cytokines/ chemokines during acute colitis. In contrast, TCDD treatment decreased IL-17, IFN-γ, MCP-1, TNF-α, KC and eotaxin-1 levels in the serum of mice with acute colitis (Fig. S1).

### AhR activation reduces both mucosal and systemic CD4^+^ CXCR3^+^ T cells after colitis

**Figure 2:**
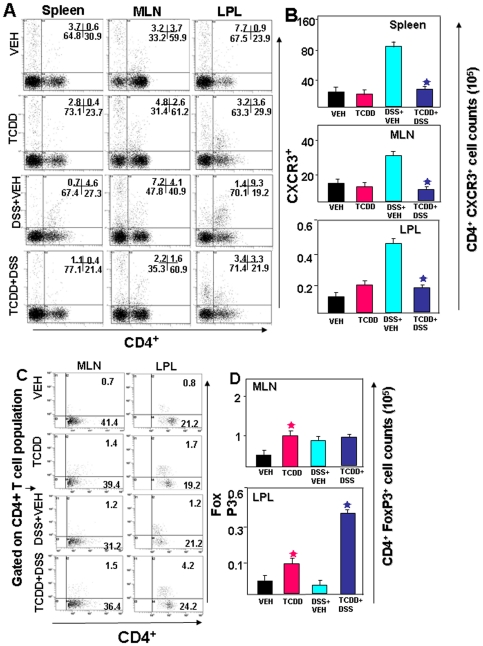
Effect of TCDD on CD4^+^ CXCR3^+^ in cells from spleen, MLN and LP and generation of Tregs in the LP following development of DSS-induced colitis. A–B. Splenic MLN and LP cells were isolated from the four groups of mice as described in Fig 1, stained for CD4^+^ CXCR3^+^ T cells, and analyzed using flow cytometry. A representative experiment is shown in panel (A), indicating the percentage of CD4^+^ CXCR3^+^ T cells, and absolute numbers (mean ± SEM) of these cells from groups of 6 mice are depicted in panel B. Asterisks (*) indicate statistically significant differences with *p*<0.01 between DSS+VEH versus DSS+TCDD treated group. C–D. MLN and LP cells were isolated from the 4 groups of mice and stained for CD4^+^ Foxp3+ cells. A representative experiment is shown in panel C, and absolute numbers (mean ± SEM) of these cells from groups of 6 mice are depicted in panel D. Asterisks (*) indicate statistically significant differences (with *p*<0.01) between VEH versus TCDD alone and DSS+VEH versus DSS+TCDD treated group.

The association between CXCR3 expression and Th1-dependent immunity has been observed in several models of inflammatory diseases. Previously we have shown that blockade of CXCR3 ligands abrogated spontaneous colitis in IL-10^-/-^ mice [35]. To examine whether TCDD differentially affected the T effector population that express CXCR3, splenic, MLN and LP cells were analyzed. We found a significant increase in the percentage of splenic, MLN and LP CD4^+^T cells that expressed CXCR3^+^ during DSS induced colitis (Fig. 2A–B), which was reversed following TCDD treatment. Moreover, the absolute number of CXCR3^+^ T cells in Spleen, MLN and LP rose significantly in DSS+vehicle groups and decreased following TCDD treatment (Fig. 2A–B).

### AhR activation triggers Treg cells during colitis

Previous studies have provided convincing evidence that Tregs play an important role in the control of intestinal inflammation [24]. Also, based on the previous report that AhR activation leads to Treg differentiation in a ligand-specific fashion [14], we next determined whether TCDD induces Tregs in DSS-induced colitis. We analyzed Tregs in the MLN and LP by flow cytometry after TCDD treatment. We noted a significant increase in the percentage and absolute numbers of Tregs in the LP in DSS+TCDD group when compared to DSS+vehicle group (Fig. 2C–D). Although a similar trend was seen in the MLN, it was not statistically significant. Also, TCDD treatment alone caused a modest increase in Tregs in both MLN and LPL when compared to vehicle controls.

### Effect of AhR activation on Foxp3 and IL-17 expression in lymphoid organs during DSS-induced colitis

**Figure 3:**
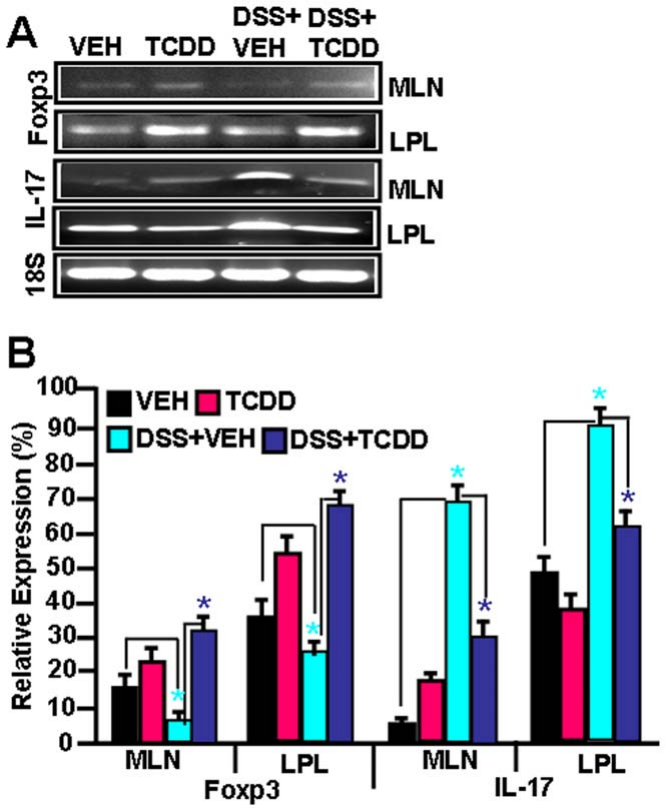
Effect of TCDD administration *in vivo* on Foxp3 and IL-17 expression in lymphoid organs during colitis. MLN, and LP cells were isolated on day 14 of colitis from the 4 groups of mice as described in Fig. 1. RT-PCR was used to detect mRNA levels for Foxp3 and IL-17 (panel A) and data from 3 independent experiments in each group is shown in panel B. Vertical bars represent mean+/− SEM of three independent experiments. Comparisons were made between VEH vs DSS+VEH groups as well as between DSS+VEH vs DSS+TCDD groups. Asterisks (*) represent statistically significant (p<0.05) difference between VEH and DSS+VEH groups and between DSS+VEH and DSS+TCDD groups.

Because of the low recovery of cells from LP, and the low frequency of Tregs and Th17 cells in the LP and MLN, we performed RT-PCR to further characterize the effect of AhR activation during colitis on induction of Tregs and IL-17. We noted a significant down-regulation of FoxP3 and upregulation of IL-17 in DSS+vehicle groups when compared to vehicle-treated normal mice (Fig. 3A–B). Following TCDD treatment of colitis (DSS+TCDD), this trend was reversed (Fig. 3A–B). We also noted that TCDD treatment into normal mice caused significant upregulation in Foxp3 expression while IL-17 levels showed no significant change, when compared to vehicle treated normal mice. The constitutive IL-17 expression in LPL in normal control mice may result from the activation of these cells by the gut flora.

### Effect of TCDD and FICZ in the differentiation of Tregs and Th17 cells in vitro

**Figure 4:**
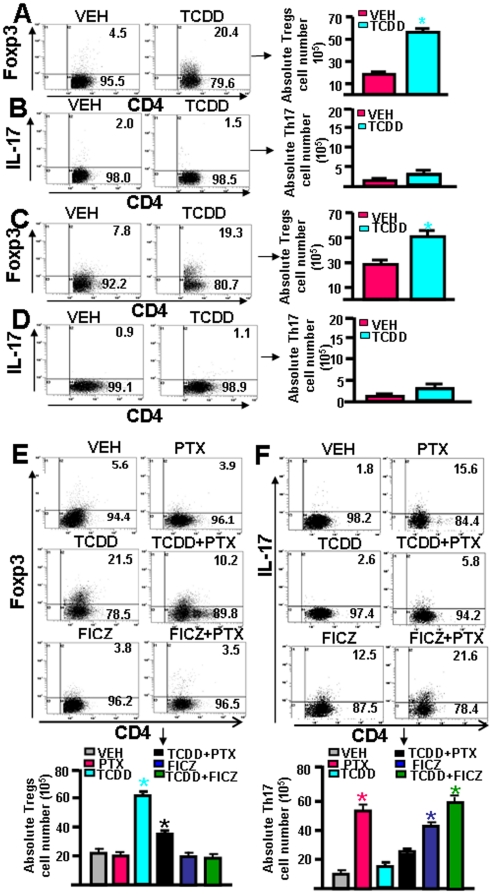
Effect of AhR activation by different ligands on reciprocal differentiation of Tregs and Th17 cells *in vitro.* T cells from naïve C57BL/6 mice were activated using anti-mouse CD3 (1 µg/ml) and CD28 (1 µg/ml) mAbs (A, B) or T cells from OTII.2a mice were activated with BMDCs pulsed with Ova peptide (C, D). The cultures received VEH or TCDD (100 nM/ml) for 72 hrs. In panels E and F, T cells from naïve C57BL/6 mice were cultured with BMDCs along with low doses of anti-CD3 Abs (0.5 µg/ml)) and pertusis toxin (PTX), to promote Th17 cells. These cultures also received VEH, TCDD (100 nM/ml) or FICZ (100 nM/ml) for 72 hrs. The cells were gated for CD4 and analyzed for Foxp3 or IL-17. Data from a representative histogram and from three independent experiments have been shown. Vertical bars represent mean ± SEM. In panels A–D, data were compared between TCDD vs VEH and in panels E and F, PTX groups were compared with PTX+TCDD. Asterisks (*) represent statistically significant (p<0.05) difference.

To further address the effect of AhR activation on Treg and Th17 differentiation, we performed a series of *in vitro* studies. T cells obtained from normal mouse MLN were activated with anti-CD3+CD28 mAbs, and treated with vehicle or TCDD. The analysis of T cells differentiation revealed that TCDD caused dramatic induction of Tregs (∼20.4%) when compared to vehicle controls (∼4.5%) (Fig. 4A). However, these culture conditions did not favor the induction of Th17 cells (Fig. 4B). Similarly, when we examined generation of Tregs and Th17 cells in Ova antigen-specific activated T cells from OT.II.2a mice, in the presence or absence of TCDD, we observed significantly higher number, and percentage of Tregs (∼19.3% CD4^+^Foxp3^+^ cells) in the presence of TCDD when compared to vehicle (∼7.8%; Fig 4C). However, these culture conditions also failed to yield significant induction of Th17 cells (Fig. 4D).

Because the culture conditions to activate T cells did not generate significant levels of Th17 cells, next we used bone marrow-derived dendritic cells (BMDCs), lower dose (0.5 µg/ml)) of anti-CD3 Abs, and pertusis toxin (PTX), which together is known to promote Th17 cells differentiation *in vitro* [36] and then tested the effect of TCDD. We also used FICZ, another AhR ligand that has been shown to promote the generation of Th17 cells but not Tregs [14]. In the presence of vehicle alone, low levels of Th17 and Tregs were generated (Fig 4E–F). However, in the presence of TCDD, there was marked induction of Tregs but not Th17 cells (Fig 4E–F). Addition of PTX to cultures lowered the generation of Tregs (Fig 4E) but increased the generation of IL-17^+^ cells (Fig 4F). Interestingly, in the presence of PTX+TCDD, differentiation of Tregs was significantly higher than in cultures with PTX alone (Fig 4E). Also, while PTX alone promoted the strong differentiation of Th17 cells, PTX+TCDD group had significantly lower percentage and numbers of Th17 cells (Fig 4F). Addition of FICZ to cultures did not cause much change in Tregs in the presence or absence of PTX (Fig 4E). However, FICZ alone caused robust differentiation of Th17 cells, which was further enhanced in the presence of PTX (Fig. 4F). Together, these data demonstrated that even in culture conditions that favor Th17 differentiation, TCDD dampens Th17 differentiation while promoting Treg differentiation. Our data suggested that AhR ligands might behave differently in as much as FICZ, under identical culture conditions, promoted Th17 differentiation but not Tregs differentiation.

### AhR plays significant role in TCDD-induced differentiation of Tregs

**Figure 5:**
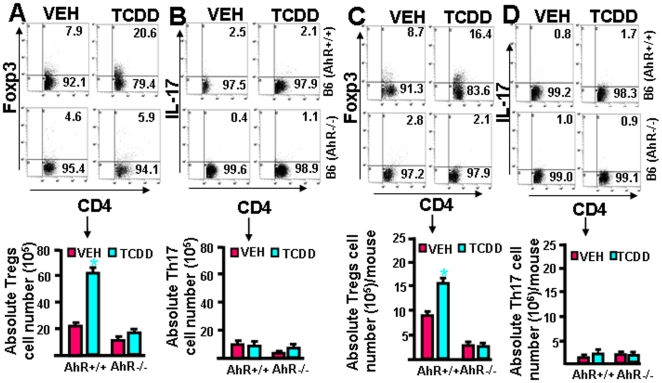
TCDD requires AhR to induce reciprocal differentiation of Tregs and Th17 cells *in vitro* and *in vivo.* In panels A and B, T cells from C57BL/6 wild-type (AhR^+/+^) and knockout (AhR^-/-^) mice were cultured in the presence of anti-mouse CD3 (2 µg/ml) and CD28 (1 µg/ml) mAbs for 72 hrs. These cells were simultaneously treated with VEH or TCDD (100 nM/ml). The cells were stained for Tregs (CD4^+^ FoxP-3^+^) and Th17 (CD4^+^ IL-17^+^) cells. In panels C and D, C57BL/6 wild type (AhR^+/+^) and knockout (AhR^-/-^) mice were injected with VEH or TCDD and 5 days later, spleen cells were analyzed for Tregs (CD4^+^ FoxP-3^+^) and Th17 (CD4^+^ IL-17^+^) cells. Both percentages and absolute numbers of Tregs and Th17 cells were depicted. Vertical bars represent data (mean ± SEM) from three independent experiments. Statistical comparisons were made between TCDD and VEH-treated groups and asterisks (*) represent statistically significant (p<0.05) differences.

To further confirm that the effects of TCDD on Treg induction was indeed mediated through AhR, we performed *in vitro* and *in vivo* experiments using wild-type C57BL/6 (AhR^+/+^) and AhR knockout (AhR^-/-^) mice. To perform *in vitro* assays, we used T cells from wild type (AhR^+/+^) and AhR knockout (AhR^-/-^) mice and activated them with anti-CD3+CD28 Abs. The cells were also treated with vehicle or TCDD (100 nM/ml). The cells were harvested on day 3 and analyzed for the expression of Foxp3 and IL-17 within the CD4^+^ T cells. As observed earlier, TCDD promoted the differentiation of Tregs in AhR^+/+^ but not AhR^-/-^mice (Fig. 5A). Also, it was noted that TCDD did not promote the differentiation of Th17 cells in the culture of T cells from either of the mice (AhR^+/+^ or AhR^-/-^; Fig. 5B). Furthermore, we performed *in vivo* experiments using wild-type (AhR^+/+^) and AhR knockout (AhR^-/-^) mice and treated them either with vehicle or TCDD. CD4^+^ T cells from spleens were examined for the presence of Tregs or IL-17^+^ Th17 cells by flow cytometry. There was significantly higher percentage and number of Tregs in wild-type mice treated with TCDD when compared to vehicle-treated mice (Fig. 5C), similar to that seen using RT-PCR analysis in other lymphoid organs (Fig 3). In contrast, TCDD failed to induce significant increase in the percentage and numbers of Tregs in the knockout (AhR^-/-^) mice (Fig. 5C). In these *in vivo* studies, we observed minimal number (<2%) of Th17 cells in the presence or absence of TCDD (Fig. 5D). These data were further corroborated by the findings of absolute numbers of Tregs and Th17 cells. Together, these data demonstrated that TCDD-mediated induction of Tregs was AhR dependent. Also, our in vivo studies indicated that TCDD could induce Tregs even in naïve mice in an AhR-dependent fashion.

### TCDD mediates partial demethylation of Foxp3 promoter and methylation of IL-17 promoter to influence their regulation

**Figure 6:**
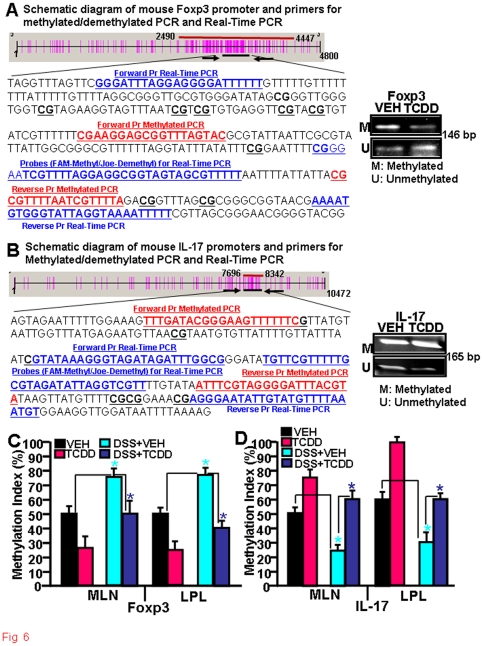
Effect of TCDD on methylation of Foxp3 and IL-17 promoters during colitis. A-B: Phyisical map generated using Methylprimer Express software (v 1.0) is demonstrating the distribution of CpG islands (vertical red bars) and the position of the MSP primer pairs on the mouse Foxp3 (A) and Il-17 (B) promoter. PCR amplicons of promoter region of Foxp3 gene amplified by methylated or demethylated sets of primers in T cells activated in vitro with anti-CD3+anti-CD28 Abs, treated in the presence of VEH or TCDD, has also been depicted in the right panels. C-D: Real Time PCR demonstrating methylation/demethylation status of Foxp3 and IL-17 promoters in MLN and LPL *in vivo*. MLNs and LP lymphocytes were isolated from the four groups of mice as described in Fig 1. Data (mean ±SEM) from 3 independent experiments are shown. Comparisons were made between VEH vs DSS+VEH groups as well as between DSS+VEH vs DSS+TCDD groups. Asterisks (*) represent statistically significant (p<0.05) difference between VEH vs DSS+VEH treated groups, and between DSS+VEH vs DSS+TCDD treated groups.

DNA methylation plays an important role in regulation of genes by silencing their expression. Because under most culture conditions, TCDD promoted Foxp3 expression, we first evaluated methylation/ demethylation status of Foxp3 gene promoter, using in vitro activated T cells from naïve C57BL/6 mice with anti-CD3+CD28 Abs in the presence of vehicle or TCDD. To this end, we performed methylated PCR (MSP) using bisulfite converted genomic DNA from T cells, using sets of mouse Foxp3-specific forward and reverse primers that could amplify methylated or demethylated region of the promoter (Fig. 6A). The data revealed lower intensity of DNA amplicon of Foxp3 in TCDD-treated T cells when methylated sets of primers were used when compared to vehicle controls, whereas the band intensity was significantly higher when demethylated sets of primers were used (Fig 6A). Similarly, we used mouse IL-17-specific forward and reverse sets of primers to amplify methylated or demethylated region of the IL-17 promoter (Fig. 6B). The data revealed higher intensity of DNA amplicon of IL-17 in both vehicle- or TCDD-treated T cells when methylated sets of primers were used but very low intensity amplicons were observed when primers to amplify demthylated region were used (Fig. 6B).

To further directly quantitate the methylated/demethylated status of Foxp3 and IL-17 gene promoters during colitis , we performed real-time PCR using sets of forward and reverse primers and Taqman probes and using bisulfite converted genomic DNA from MLN or LP cells of the following groups of mice: Vehicle alone, TCDD alone, DSS+vehicle, and DSS+TCDD. MLN and LP cells from mice treated with DSS+vehicle showed higher level of methylation (∼75%) for Foxp3 promoter (Fig. 6C) but lower levels of methylation (∼25% for MLN and ∼30% for LPL) for IL-17 promoter (Fig. 6D) when compared to vehicle-treated control mice (Fig. 6C). Interestingly, this process was reversed in DSS+TCDD treated groups when compared to DSS+Veh exposed mice in both the MLN and LPL cells. Also, analysis of methylation status of Foxp3 and IL-17 promoters in MLN or LPL cells from mice treated with TCDD alone when compared to vehicle-treated mice, showed an overall decreased methylation of Foxp3 and enhanced methylation of IL-17 promoters. These data supported the observation that during DSS-induced colitis, hypermethylation of Foxp3 promoter and hypomethylation of IL-17 promoter may lead to decreased expression of Foxp3 and increased induction of IL-17. Furthermore, TCDD treatment may reverse this process thereby ameliorating the colitis.

## Discussion

A large number of environmental chemicals and other xenobiotics that enter the body mediate their influence on cellular functions by either nonselective binding to cellular macromolecules or specifically binding to cellular receptors. One of these intracellular chemosensor molecules is the aryl hydrocarbon receptor (AhR), a transcription factor of the bHLH/PAS family that is known to regulate the biochemical and toxic effects of dioxins, polycyclic aromatic hydrocarbons, and structurally related compounds [37]. Recent studies have identified endogenous ligands for AhR and suggested that AhR is not only a master regulator of drug metabolism but also regulates many physiological functions such as cell growth and differentiation. More recently, AhR has been shown to regulate the differentiation of T cells and therefore play a critical role in the modulation of immune response. Because environmental chemicals are well known to modulate the immune system [38], it is also likely that the above studies help establish a link between environmental contaminants and their ability to alter T cell differentiation through activation of AhR [39]. In this context, the current study demonstrates, using TCDD, how AhR activation leads to selective upregulation of FoxP3^+^ Tregs and down regulation of Th17 cells through epigenetic regulation, leading to amelioration of an inflammatory disease such as colitis.

In this study, we examined the effect of AhR activation on DSS-induced colitis in the mouse model. We observed that TCDD administration reversed DSS-induced colitis in mice. A single dose of TCDD treatment blocked colitis symptoms, corrected weight loss, and reduced systemic expression of IL-17, IFN-γ, MCP-1, exotaxin-1, and TNF-α levels. Our results also suggested that the increase in the number of mucosal CXCR3^+^ T cells seen during acute colitis could be mitigated by TCDD treatment. Moreover, TCDD elevated Foxp3 expression in the MLN and LP of mice with colitis. Taken together, these results suggested that TCDD ameliorates acute colitis by increased generation of Tregs in the LP of mice with colitis, with consequent decrease in CXCR3^+^ T cell expression.

CXCR3 is express by activated T, epithelial and lymphoid derived cells. CXCR3 ligands, such as CXCL9 and CXCL10, are upregulated in many inflammatory diseases and we have reported that the CXCR3 ligands are upregulated during IBD specifically [40]. We have also shown that antibody-mediated neutralization of the CXCR3 ligand CXCL10 inhibits chronic colitis in IL-10^-/-^ mice [35]. CXCR3 ligands mainly attract activated T cells of the Th1 phenotype that express high levels of CXCR3 [41]. In addition to the pathogenic role played by Th17 cells during colitis [30], it is also known that colitis may be mediated by cytokine-producing Th1 cells that infiltrate the mucosa [42]. Such studies stress the importance of CXCR3 and its ligands in colitis development [32]. In the present study, we noted reduced numbers of CXCR3^+^ T cells in the spleen, MLN and LP after TCDD treatment, which may result from direct inhibition of such cells or indirectly through induction of Tregs, which may result in the amelioration of chronic colitis.

TCDD is well known for its immunosuppressive effects, specifically causing thymic atrophy and suppression of a wide range of immune functions [43]. While the ability of TCDD to cause immunosuppression has been widely reported, the precise mechanisms have been widely debated. In addition to the direct effect on the differentiation of Tregs and Th17 cells, AhR activation may increase prostaglandin E2 production in the colon, which may lead to amelioration of colitis [44]. It is interesting to note that AhR-dependent activation of suppressor T cells had been characterized by one of us as a potential mechanism of TCDD-induced immunosuppression almost 30 years ago [45], [46], at a time when FoxP3^+^ Tregs had not been discovered. In addition, studies from our laboratory, subsequently, also demonstrated that TCDD was able to induce apoptosis in activated T cells leading to suppression of the immune functions [15], [16], [17], [47]. This stems from the fact that, TCDD can induce the expression of AhR, Fas and FasL and trigger the extrinsic pathway of apoptosis in activated T cells [15], [16], [48]. We also noted that TCDD regulates Fas and FasL promoters through dioxin responsive element (DRE) and/or NF-kappaB motifs [48]. While recent studies have found conflicting results on the induction of Th17 or Tregs and have attributed this to the affinity of AhR ligands [14], none of these studies have taken into account the potential role of TCDD-induced apoptosis in activated T cells, which may influence the nature of T cells generated and the effector functions. Thus, clearly additional studies are necessary to determine if TCDD can selectively induce differential levels of apoptosis in activated Th17 and Tregs to mediate immunosuppressive effects.

The fact that AhR activation leads to suppression of inflammation has been used recently to demonstrate its efficacy against some experimental models of inflammation and autoimmune disease. For example, TCDD was shown to suppress diabetes in NOD mice by potentially increasing Foxp3+ T cells in pancreatic lymph nodes [49]. Also, TCDD inhibited the clinical symptoms in an experimental autoimmune encephalomyelitis (EAE) model by inducing Tregs [14]. Interestingly, in the same study, 6-formylindolo [3,2-b] carbazole (FICZ) interfered with Treg cell development, and boosted Th17 cell differentiation thereby increasing the severity of EAE. These findings are consistent with the current study wherein we noted that while TCDD increased Tregs and suppressed Th17 cells in culture, FICZ had opposite effects. These studies suggest that AhR may regulate both Treg/Th17 differentiation in a ligand-specific fashion, and therefore the nature of the ligand and the dose may play a critical role while determining the potential for therapeutic intervention. It is also likely that different subsets of T cells may exhibit differential levels of sensitivity to apoptosis following AhR activation, an aspect that needs to be evaluated further.

Epigenetic modification by CpG methylation at specific sites in the promoters of various genes present in T cells is getting increased scrutiny as mechanism of regulation of T cell differentiation into various subsets [50], [51], [52]. Both Foxp3 and IL-17 promoters possess CpG islands in their promoter regions. Thus, we examined the methylation status of CpG islands present in Foxp3 and IL-17 promoters following AhR activation. We selected the promoter regions that carry the maximum number of CpG islands in Foxp3 and IL-17, to examine their methylation/demethylation status. As shown in Figure 9, we observed demethylation of CpG islands present in the Foxp3 promoter and increased methylation of CpG islands of the IL-17 promoter, following activation of AhR during colitis. These data suggested that AhR activation by TCDD may initiate signaling leading to demethylation or methylation of Foxp3 and IL-17 genes. It has been reported that demethylation induced by 5-Aza-2′-deoxycytidine in human NK cells leads to Foxp3 expression [53]. In another report, Kim and Leonard demonstrated that 10% to 45% of the CpG sites in the Foxp3 proximal promoter are methylated in naive CD4^+^CD25^−^ T cells, whereas all CpG sites were demethylated in natural Tregs [54]. They also reported that TGF-β induced demethylation of CpG in CD4^+^CD25^−^ T cells [54]. In another study, it has been shown that CpG regions of Foxp3 is approximately 70% methylated in CD4^+^CD25^lo^ cells compared with approximately 5% in CD4^+^CD25^hi^ T cells in humans [55]. These studies demonstrate that methylation of the proximal promoter is an important regulator of Foxp3 expression. In another study, it was noted that Th17 cell differentiation was accompanied by epigenetic changes at IL-17 gene promoter and suggested that similar to other T helper cell lineages, epigenetic modification is an integral part of Th17 cell differentiation [56]. They observed hyperacetylation of histone H3 at the IL-17 promoter, and proposed the role of IL-6 and TGF-â in dictating epigenetic modification of IL-17 gene [12].

The precise mechanisms through which AhR activation by TCDD leads to epigenetic regulation is unclear and there are very few investigations in this area. In one study, the authors tested why TCDD would induce both CYP1A1 and CYPIB1 in human MCF-7 cells, while in HepG2 cells, only CYP1A1 was inducible [57]. They found that the deficiency of CYP1B1 induction in HepG2 cells was due to cytosine methylation at the promoter CpG dinucleotides. Thus, treatment of HepG2 cells with the DNA methyl transferase inhibitor partially demethylated the CpG dinucleotides in the *CYP1B1* gene promoter and restored TCDD-mediated induction of CYP1B1. In another study, the effect of postnatal exposure to a reconstituted mixture of AhR agonists was studied in rats, specifically on DNA methyl transferases [58]. AhR activation caused reduction in DNA methyltransferase-1 (*Dnmt1*) mRNA to 28% and 32% of control, in the liver and hypothalamus, respectively. Because DNA methyltransferases are responsible for the generation of genomic methylation patterns leading to gene silencing, the authors suggested that early post-natal exposure to environmental pollutants that act as AhR ligands could cause changes in DNA methylation thereby impacting gene expression [58].

In summary, our studies suggest that during colitis, activation of AhR by TCDD may promote anti-inflammatory activity primarily through epigenetic regulation of Foxp3 and IL-17 gene promoters leading to preferential differentiation of Tregs and inhibition of Th17 cells. The Tregs, in turn, may also suppress DSS-induced Th1 cells and CXCR3^+^ T cells to emigrate from MLN to the LP and inhibit cytokine/chemokine production such as TNF-α, MCP-1, IL-17, IFN-γ and Eotaxin-1, leading to attenuation of acute colitis.

## Materials and Methods

### Animals

Female C57BL/6 mice aged 8 to 12 weeks were purchased from Jackson Laboratories (Bar Harbor, ME).

### Ethics Statement

The mice were housed and maintained in micro-isolator cages under conventional housing conditions at the AAALAC accredited South Carolina School of Medicine animal facility. Experimental groups consisted of six mice each and the study was repeated three times.

### Acute colitis induced by DSS and TCDD treatment

Acute colitis was induced using DSS as described elsewhere [31]. Briefly, eight week-old C57BL/6 mice received either water or drinking water containing 3% DSS (MP Biomedical, LLC, Ohio) (*ad libitum)* for seven days followed by water cycle alone for seven days. The body weight of mice was monitored every day from day 0 at the start of TCDD treatment. TCDD was a generous gift from Dr. Chae (Research Triangle Park NC). TCDD was dissolved in corn oil and groups of six mice received either 100 µl of 25 µg/kg body weight single dose of TCDD by intra peritoneal (i.p.) injection as described [59] or vehicle on the first day of DSS exposure. At the end of the experiment, blood and colon samples were collected. The colon was washed with phosphate-buffered saline, cut longitudinally, formalin fixed, and paraffin embedded.

### Cell isolation

Spleens and mesenteric lymph nodes (MLN) from individual mice were mechanically dissociated and RBCs were lysed with lysis buffer (Sigma St. Louis, MO). Single cell suspensions of spleen and MLN were passed through a sterile wire screen (Sigma St. Louis, MO). Cell suspensions were washed twice in RPMI 1640 (Sigma St. Louis, MO) and stored in media containing 10% fetal bovine serum (FBS) on ice until used after one to two hours. The small intestine/colon was cut into 1-cm stripes and stirred in PBS containing 1 mM EDTA at 37°C for 30 min. The cells from intestinal lamina propria (LP) cells were isolated as described previously [35]. In brief, the LP was isolated by digesting intestinal tissue with collagenase type IV (Sigma St. Louis, MO) in RPMI 1640 (collagenase solution) for 45 min at 37°C with moderate stirring. After each 45 min interval, the released cells were centrifuged, stored in complete medium and mucosal pieces were replaced with fresh collagenase solution for at least two times. LP cells were further purified using a discontinuous Percoll (Pharmacia, Uppsala, Sweden) gradient collecting at the 40–75% interface. Lymphocytes were maintained in complete medium, which consisted of RPMI 1640 supplemented with 10 ml/L of nonessential amino acids (Mediatech, Washington, DC), 1 mM sodium pyruvate (Sigma), 10 mM HEPES (Mediatech), 100 U/ml penicillin, 100 µg/ml streptomycin, 40 µg/ml gentamycin (Elkins-Sinn, Inc., Cherry Hill, NJ), 50 µM mercaptoethanol (Sigma) and 10 % FCS (Atlanta Biologicals).

### Flow cytometry analysis

Cells from the spleen, MLN, and LP were freshly isolated as described above for each experimental group. For three to four color FACS cell surface antigens staining, cells were pre-blocked with Fc receptors for 15 min at 4°C. The cells were washed with FACS staining buffer (PBS with 1% BSA), and then stained with CY-, FITC- or PE-conjugated anti-CD4 (H129.19) (BD-PharMingen, San Diego CA), and/or–CXCR3 (CXCR3-173), FoxP3 (FJK-16s) (ebioscience, San Diego CA), for 30 minutes with occasional shaking at 4°C. The cells were washed twice with FACS staining buffer and thoroughly re-suspend in BD Cytofix/Cytoperm (BD-PharMingen, San Diego CA) solution for 20 min. The cells were again washed twice with BD perm/wash solution after keeping it for 10 min at 4°C. Lymphocytes were then washed thoroughly with FACS staining buffer and analyzed by flow cytometry (FC 500 by Beckman Coulter Fort Collins Co).

### Cytokine quantitation by Luminex™ analysis

T helper cell-derived cytokines, IL-6, IL-17, TNF-α, MCP-1, IFN-γ, and Eotaxin-1 in the serum were determined by a luminex Elisa assay kit (Millipore Corporation, MA USA). IL-6, TNF-α, MCP-1, IL-17, IFN-γ and Eotaxin-1 analyte beads in assay buffer were added into pre-wet vacuumed wells followed by 25 µl of serum or standard solution, 25 µl of assay buffer, and 25 µl of assay beads, and incubated overnight at 4°C with continuous shaking (at setting #3) using a Lab-Line™ Instrument Titer Plate Shaker (Melrose, IL). The filter bottom plates were washed as before and centrifuged at 300x g for 30 seconds. Subsequently, 25 µl of anti-mouse detection Ab was added to each well and incubated for 1 hour at room temperature. Next, 25 µl streptavidin-phycoerythrin solution was added and incubated with continuous shaking for 30 minute at room temperature. 200 µl of wash buffer was added and Milliplex™ readings were measured using a Luminex™ System (Austin, TX) and calculated using Millipore software. The Ab Milliplex™ MAP assays were capable of detecting >10 pg/ml for each analyte.

### Histology

Colon was preserved using 10% buffer neutral formalin followed by 4% paraformaldehyde and embedded in paraffin. Fixed tissues were sectioned at 6 µm, and stained with hematoxylin and eosin for microscopic examination. Intestinal lesions were multi-focal and of variable severity. Grades were given to intestinal sections that took into account the number of lesions as well as severity. A score (0 to 12) was given based on the established criteria already described [35]. The summation of these scores provided a total colonic disease score per mouse. The summation of these disease scores provided a total colonic disease score that could range from 0 to 12 with grade 1 lesions in proximal, middle and distal colon segments.

### In vitro differentiation of Tregs and Th17 cells in the presence of TCDD

To determine TCDD-induced differentiation of Tregs and Th17 cells *in vitro*, we performed a series of *in vitro* assays. In brief, lymphocytes from C57BL/6 mice were cultured in the presence of purified anti-mouse anti-CD3 (2 µg/ml) and anti-CD28 (1 µg/ml) mAb for 3 days. These cells were also simultaneously treated with vehicle (DMSO) or TCDD (100 nM/ml). On day 3, the cells were further cultured in the presence of PMA (10 ng/ml) and Ionomycin (1 µg/ml) for 5–6 hrs and in last four hrs of culture, Golgi stop was added into the culture. The cells were then harvested, washed twice with PBS, and analyzed for the presence of Tregs and Th17 cells in the culture. Intracellular staining of cells was performed using BD Cytofix/Cytosperm kit using their protocol (BD Biosciences, San Diego, CA). FITC-labeled anti-mouse CD4 and PE-labeled anti-mouse Foxp3 mAbs were used to detect Tregs, and FITC-labeled anti-mouse CD4 and PE-labeled anti-mouse IL-17 mAbs, to detect IL-17 positive Th17 cells.

Differentiation of Tregs and Th17 cells in antigen-specific activation of T cells was also investigated in the presence or absence of TCDD. To this end, T cells from OT.II.2a mice were cultured in the presence of bone marrow-derived dendritic cells (BMDCs) from C57BL/6 mice. BMDCs were generated as described earlier [59]. In brief, T cells from OT.II.2a mice were cultured in the presence of Ova peptide (OvaPep)-pulsed BMDCs and with vehicle or TCDD for 3 days. Three days post culture, the cells were harvested and the presence of Tregs and IL-17 positive Th17 cells in the culture was determined as described above.

TCDD-induced differentiation of Tregs and Th17 cells was also examined in the absence or presence of Pertussis toxin (PTX), a culture condition that promotes Th17 differentiation [36]. To this end, we performed *in vitro* assays using T cells from C57BL/6 mice that were cultured in the presence of matured BMDCs from C57BL/6 mice and CD3 (0.5 µg/ml) Ab for 3 days. These cells were simultaneously treated with or vehicle or PTX (5 µg/ml), TCDD (100 nM/ml), TCDD (100 nM/ml) + PTX (5 µg/ml), 6-formylindolo [3,2-b] carbazole (FICZ) (100 nM/ml), and FICZ (100 nM/ml) + PTX (1 µg and 5 µg/ml). Three days post culture, the cells were harvested, washed twice with cold PBS and intracellular staining was performed as described earlier. Anti-mouse CD4-FITC and Foxp3-PE mAbs were used to detect Tregs and anti-mouse CD4-FITC and IL-17-PE mAbs were used to detect IL-17 positive Th17 cells. The cells were analyzed by flow cytometry.

### Role of AhR in TCDD-induced differentiation of Tregs and Th17 cells

To investigate the role of AhR in TCDD-induced differentiation of Tregs and Th17 cells, we used wild type C57BL/6 (AhR^+/+^) and AhR knockout (AhR^-/-^) mice. The mice were injected vehicle or TCDD intraperitoneally (ip) and on day 6, spleens from treated mice were harvested, single cell suspension prepared, and the cells were cultured in the presence of PMA (10 ng/ml) and Ionomycin (1 µg/ml) for 5–6 hrs and in last four hrs of culture, Golgi stop was added into the culture. Staining of cells was performed using anti-mouse CD4-FITC and Foxp3-PE for Tregs, and anti-mouse CD4-FITC and IL-17-PE for IL-17^+^ T cell population. The cells were later analyzed by flow cytometry.

To further understand the role of AhR in TCDD-induced differentiation of Tregs and Th17 cells, a series of *in vitro* assays were performed. In brief, lymphocytes from C57BL/6 (AhR^+/+^) and AhR ^-/-^ mice were cultured in the presence of anti-CD3 (2 µg/ml) and anti-CD28 (1 µg/ml) Abs for 3 days. These cells were simultaneously treated with vehicle or TCDD (100 nM/ml). On day 3, the cells were further cultured in the presence of PMA (10 ng/ml) and Ionomycin (1 µg/ml) for 5–6 hrs and in last 4 hrs of culture, Golgi stop was added into the culture. Cells were harvested, washed twice with cold PBS, and staining of the cells was performed using anti-mouse CD4-FITC and Foxp3-PE mAbs for Tregs and anti-mouse CD4-FITC and IL-17-PE for IL-17^+^ Th17 cells followed by flow cytometric analysis.

### Reverse Transcriptase PCR (RT-PCR) to determine the expression of Foxp3 and IL-17 in T cells in the presence or absence of TCDD

First strand cDNA synthesis was performed using total RNA (1 µg) isolated from T cells cultured in the presence of purified anti-mouse anti-CD3 and anti-CD28 mAbs as described earlier and treated with vehicle (DMSO) or TCDD for 3 days using iScript Kit and following the protocol of the manufacturer (Bio-Rad). To detect the expression Foxp3 and IL-17, sets of primers specific to mouse Foxp3 and IL-17 were used. PCR was performed as described earlier [48]. The PCR products, generated from mouse Foxp3 and IL-17 primer pairs, were normalized against PCR products generated from mouse 18S forward (5′-GCCCGAGCCGCCTGGATAC-3′) and reverse (5′-CCGGCGGGTCATGGGA ATAAC-3′) primers after electrophoresis on 1.5% agarose gel and visualization with UV light. The band intensity of PCR products was determined using BioRad image analysis system (BioRad, Hercules, CA).

### Detection of methylation in promoters of Foxp3 and IL-17 genes

We examined methylation/demethylation of Foxp3 and IL-17 gene promoters in T cells in the presence or absence of TCDD or in various samples harvested from mice exposed to DSS and treated with vehicle or TCDD. To examine TCDD-induced regulation of Foxp3 and IL-17 genes, total genomic DNA from T cells treated with vehicle or TCDD was isolated using DNeasy Blood & Tissue kit from Qiagen and following the protocol of the company (Qiagen). Bisulfite modification of total DNA was performed using Bisulfitization kit from Qiagen. The DNA concentration was measured using spectrophotometer. Purified DNA, post bisulfitization, was either used immediately or stored at −20°C for future use.

### Methylated PCR

To amplify methylated or demethylated regions of mouse Foxp3 promoter, PCR was performed using mouse bisulfite converted genomic DNA and mouse Foxp3-specific pair of forward (5′-CGAA GGAGCGGTTTAGTAC-3′) and reverse (5′-TCTAAAACGATTAAAACGCG-3′ ) primers to amplify methylated region or a pair of forward (5′-TTTTGAAGGAGTGGTTTAGTAT-3′ ) and reverse (5′-CCATCTAAAACAATTAAAACACA-3′) primers to amplify demethylated region. Similarly, to amplify methylated or demethylated regions of IL-17 promoter, genomic PCR analysis was performed using mouse IL-17-specific pair of forward (5′-TTTGATACGGGAAGTTTTTTC-3′) and reverse (5′-TACGTAAATCCCCTACGAAAT-3′) primers to amplify methylated region or a pair of forward (5′-AAGT TTGATATGGGAAGTTTTTTT-3′) and reverse (5′-TACATAAATCCCCTACAAAATTA-3′) primers to amplify demethylated region. PCR reactions was carried out in a volume of 25 µl containing 1X EpiTect master mix (EpiTect MSP PCR kit, Qiagen)), bisulfite-converted DNA (500 ng), and primer pairs (0.3–0.4 µM). Amplification was performed in a thermal cycler (BioRad) using the following profile: one step at 95°C for 10 min, three steps cycling; 45 cycles at 94°C for 15 sec, 58°C for 30 sec, and 72°C for 45 sec, and one step at 72°C for 10 min. The PCR products, generated from mouse Foxp3 or IL-17 primer pairs were detected by electrophoresis using 2% agarose gel and visualization with UV light. The band intensity of PCR products was determined using BioRad image analysis system (BioRad).

### Real-Time methylated PCR

Real-Time methylated PCRs to detect methylation/demethylation in the promoters of Foxp3 and IL-17 genes containing CpG islands were performed using EpiTect MethyLight kit from Qiagen following the protocol of the company. To this end, Real-Time PCRs were performed using bisulfite converted genomic DNA and pairs of mouse Foxp3-specific forward (5′-GGGATTTAGGAGGGGATTT TTT-3′) and reverse (5′-GAAAAATTTTACCTAATACCCACATTTT-3′ ) and IL-17-specific forward (5′-GTATAAAGGGTAGATAGATTTGG-3′) and reverse (5′-ACATTTAAAACATACAATATTCC CT-3′) sets of primer pairs and TaqMan hybridization probes (methylated Foxp3 probe: FAM-5′-TCGT TTTAGGAGGCGGAGTAGCGTTTTT-3′, demethylated Foxp3 Probe: Joe-5′-TTGTTTTAGGAGGT GGTA GTAGTGTTTTT-3′, methylated IL-17 probe: FAM-5′-TGTTCGTTTTTGCGTAGATATTAG GTC GTT-3′ and demethylated IL-17 probe: Joe-5′-TTTGGTGGGATATGTTTGTTTTTGTG T-3′). Real-Time methylated PCR reactions were carried out in a volume of 25 µl using EpiTect MethyLight master Mix (Qiagen), 500 ng bisulfite converted genomic DNA, and pairs of forward and reverse primers (100nM) and TaqMan probes (50 nM). Amplification and detection was carried out using the following profile: one step at 95°C for 5 min, and 45 cycles at 95°C for 15 sec and 58°C for 1 min. The methylation index (%) of each sample was calculated using the following equation: Methylation index  =  M/M+U x 100%, where M is the quantity of methylated and U is the quantity of demethylated Foxp3 or IL17 real-time MSP following bisulfite conversion. The samples were categorized as demethylated, low methylation (1%–50%), or high methylation (51%–100%). All samples were run in triplicate and the average values were used.

### Statistics

The data were expressed as the mean ± SEM and compared using a two-tailed paired Student's *t*-test or an unpaired Mann Whitney U test. The results were analyzed using the Statview II statistical program (Abacus Concepts, Inc., Berkeley, CA) and Microsoft Excel (Microsoft, Seattle, WA). Single-factor variance ANOVA analyses were used to evaluate groups. Results were considered statistically significant if *p* values were <0.05 between the control and the experimental groups.

## Supporting Information

Figure S1 **TCDD mediated reduction of serum cytokines and chemokines in DSS- induced colitis.** Colitis was induced in mice that were exposed to VEH or 25 µg /kg body weight of TCDD as described in the legend to Fig 1. Serum cytokines and chemokines were measured 14 days after the DSS induction of colitis by ELISA assay. The data presented are the mean concentrations from 6 mice ± SEM in serum. Asterisks indicate statistically significant differences; i.e., *p*<0.01 between DSS+VEH versus DSS+TCDD treated group.
(TIF)
