# Supplementary figures and images for: Activation of Aryl Hydrocarbon Receptor (AhR) Leads to Reciprocal Epigenetic Regulation of FoxP3 and IL-17 Expression and Amelioration of Experimental Colitis
Source: PLoS One. 2011 Aug 15;6(8):e23522. doi: 10.1371/journal.pone.0023522 (PMC3156147; doi:10.1371/journal.pone.0023522)

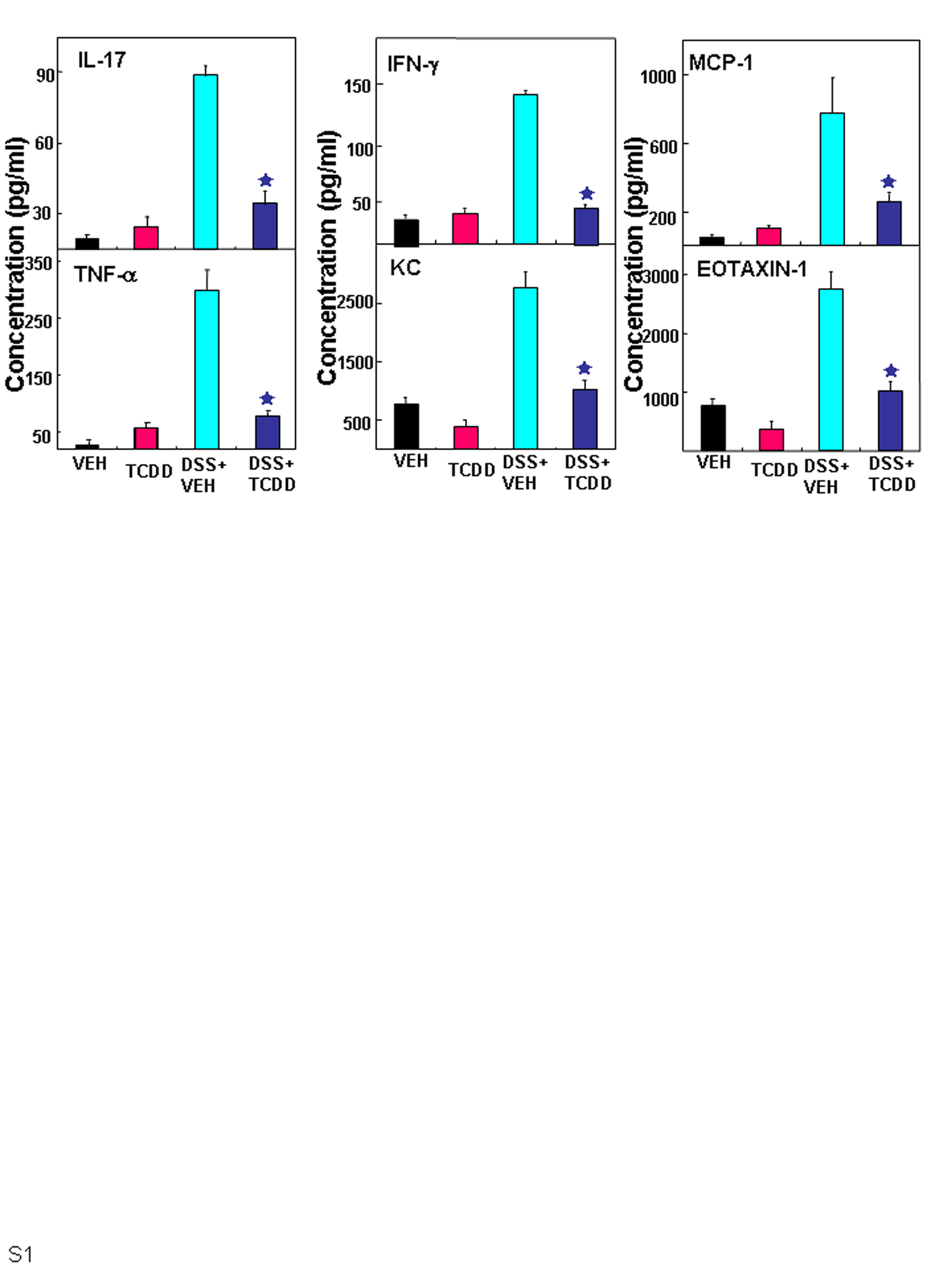

Supplement: Figure S1 — TCDD mediated reduction of serum cytokines and chemokines in DSS- induced colitis. Colitis was induced in mice that were exposed to VEH or 25 µg /kg body weight of TCDD as described in the legend to Fig 1. Serum cytokines and chemokines were measured 14 days after the DSS induction of colitis by ELISA assay. The data presented are the mean concentrations from 6 mice ± SEM in serum. Asterisks indicate statistically significant differences; i.e., p<0.01 between DSS+VEH versus DSS+TCDD treated group. (TIF) [file pone.0023522.s001.tif]
